# Relationship between serum thyroid hormone and interleukin-1b levels and postmortem tissue deiodinase activity in critically ill patients

**DOI:** 10.5937/jomb0-46999

**Published:** 2024-09-06

**Authors:** Zhenzhou Zhong, Xiaoliu Xiao

**Affiliations:** 1 Ganzhou People's Hospital, Department of Emergency, Ganzhou City, JiangXi Province, China

**Keywords:** critical illness, prognostic markers, thyroid hormone, IL-1b, deiodinase, kritična bolest, prognostički markeri, hormon štitne žlezde, IL-1b, dejodinaza

## Abstract

**Background:**

This study investigated the relationship between serum thyroid hormones and interleukin-1b (IL-1β) levels and postmortem tissue deiodinase activity in critically ill patients.

**Methods:**

Serum thyroid hormones and IL-1β were measured on the 5th, 15th, and last day of 80 critically ill patients. Forty of these patients were non-survived, and liver and skeletal muscle were harvested to analyze type 1, 2, and 3 iodothyronine deiodinases (D1, D2, and D3) activity.

## Abstract (sr)

**Uvod:**

Cilj ovog istraživanja je bio da se ispita odnos izme|u nivoa serumskih hormona štitne žlezde i interleukina-1b (IL-1b) i aktivnosti dejodinaze postmortem tkiva kod pacijenata u kritičnom stanju.

**Metode:**

Nivoi serumskih hormona štitne žlezde i IL-1b su mereni petog, petnaestog i poslednjeg dana kod 80 pacijenata u kritičnom stanju. Četrdeset od ovih pacijenata nije preživelo, a jetra i skeletni mišići su uzeti za analizu aktivnosti jodotironin dejodinaza tipa 1, 2 i 3 (D1, D2 i D3).

**Rezultati:**

Nivoi serumskog hormona koji stimuliše štitnu žlezdu (TSH), tetrajodotironina (T4) i trijodotironina (T3) su bili smanjeni, dok su reverzni trijodotironin (rT3) i IL-1b bili povećani kod pacijenata koji nisu preživeli. Od petog do poslednjeg dana, nivoi serumskog TSH, T4 i T3 su se povećavali, a nivoi rT3 i IL-1b su se vremenom smanjivali kod preživelih, dok su nivoi seruma TSH, T4 i T3 opadali ili ostajali nepromenjeni. S druge strane, rT3 i IL-1b su se povećavali kod onih koji nisu preživeli. Poslednjeg dana, aktivnost jetrenog D1 je bila u negativnoj korelaciji sa serumskim rT3 i IL-1b, dok su aktivnosti D3 jetre i skeletnih mišića bile u pozitivnoj korelaciji.

**Zaključak:**

Nivoi serumskih hormona štitne žlezde i IL-1b su povezani sa aktivnošću dejodinaze postmortem tkiva kod pacijenata u kritičnom stanju.

## Introduction

Critical illness causes hormonal changes that differ between acute and long-term illnesses [1]. Inacute critical illness, serum catecholamines, growth hormone, and cortisol levels are elevated, insulin resistance is reduced, and low triiodothyronine (T3) syndrome is observed [2]. In long-term critical illness, catecholamine and cortisol levels are reduced, and thyroid stimulating hormone (TSH) and thyroid hormone levels are reduced compared to acute cases. Although no evidence has indicated the harm of acute changes, it is unclear whether endocrine changes in long-term critical illness are all beneficial, as studies have shown that some changes may lead to clinical deterioration [3].

High-dose growth hormone, glucocorticoid, or thyroid hormone have no or even negative effects on clinical outcomes of critically ill patients [4] [5] [6] [7]. Intervening with the hypothalamic releasing factor may be possible to restore pulsating secretion of pituitary hormones and normalize peripheral hormone levels [8].

A decrease in serum T3 concentration in critical illness and an increase in serum reverse triiodothyronine (rT3) are associated with the severity of the disease [9]. Type 1, 2, and 3 iodothyronine deiodinases (D1-3) mediate peripheral thyroid hormone metabolism. Specifically, D1 mediates the formation of serum T3 from tetraiodothyronine (T4) and metabolite rT3 breakdown D2 converts T4 to T3 through outer ring deiodination and is important for local T3 production, while D3 catalyzes the inactivation of T4 and T3, generating rT3 and 3,3‘-T2 [10] [11]. D1, D2, or both reduce the deiodination of peripheral T4, significantly reducing circulating T3 levels [12] [13]. D1 is the main pathway of rT3 clearance, and this mechanism may explain the increase in serum rT3 levels [14]. In addition to D1 activity reduction, impairment in transporting T4 and rT3 to D1-containing tissues may be another mechanism of thyroid hormone disorders [15]. However, the possibility that increased D3 activity leads to decreased serum T3 levels and elevated rT3 levels should also be considered [16].

Critical illness has been shown to be associated with disturbed metabolic and inflammatory responses [17]. Interleukin-1β (IL-1β) is a pro-inflammatory cytokine that is higher in non-survivors than in critically surviving patients [18]. Other studies have shown that IL-1β can cause damage or apoptosis of thyroid follicular cells and promote the onset of autoimmune thyroiditis [19] [20] [21]. IL-1β is involved in autoimmune thyroiditis by inducing intercellular adhesion molecule-1 on thyroid follicular cells and interfering with the integrity of thyroid epithelium [22] [23]. More importantly, postoperative serum IL-1β levels in critically ill patients after major abdominal surgery are associated with mortality [24]. In addition, it has been shown that IL-1β inhibits thyroid hormone receptor-β1 gene expression [25], which inhibits hepatic D1 expression [26]. Therefore, we chose IL-1β as the focus of our study to investigate its relationship with prognosis and deiodinase activity in critically ill patients.

In short, the study observed serum thyroid hormones and IL-1β in critically ill patients and analyzed the correlation between deiodinase activity with serum thyroid hormones and IL-1β.

## Materials and methods

### Patients

Eighty patients who were hospitalized for more than 5 days in an intensive care unit (ICU) were included in this analysis. Among them, there were 39 patients with cardiac surgery, 17 patients with complex surgery (defined as patients with complications after abdominopelvic surgery, lung or esophageal surgery, or vascular surgery), 9 patients with organ transplantation, 7 patients with trauma, burn, or prosthetic surgery, and 8 patients with other surgeries. Blood samples were taken on the 5^th^, 15^th^, and last day after admission to ICU. Forty patients did not survive, and liver and skeletal muscle (rectus abdominis) were obtained within minutes of death.

### Serum analysis

Treatment of ICU patients often includes systemic or local infusions of heparin to prevent vascular coagulation, which largely affects the determination of serum-free thyroid hormones [27]. Heparin can lead to falsely elevated free thyroid hormone results. Specifically, heparin can activate lipoprotein esterase *in vitro* to release free fatty acids, which can displace bound thyroid hormone from thyroxine-binding globulin, resulting in falsely elevated free thyroid hormone levels [27] [28] [29]. Therefore, the determination of serum-free T4 and T3 was avoided. Vitros ECi Immuno diagnostic System (Ortho-Clinical Diagnostics) tested serum total T4, total T3, and TSH. rT3 was measured by radioimmunoassay [30]. IL-1β detected serum IL-1β levels in an enzyme-linked immunosorbent assay kit (R&D Systems, USA). Normal TSH, T4, T3, and rT3 values in 80 healthy subjects were measured.

### Peripheral blood mononuclear cells (PBMCs)

Whole blood (2 mL) was equally diluted with phosphate-buffered saline (PBS) and transferred into a centrifuge tube containing 3 mL Ficoll Paque (G.E. Healthcare). PBMCs were collected after centrifugation at 400×g for 20 min, rinsed twice in 10 mL PBS, and re-suspended in a lysis buffer for protein extraction.

### Immunoblotting

PBMCs and loading buffer (Yeasen, Shanghai, China) were heated at 99 °C for 10 min. Protein was loaded onto the sodium dodecyl sulfate-polyacrylamide gel and then imprinted onto the polyvinylidene fluoride membrane. The membrane was incubated overnight with either IL-1β (1:1000, R&D Systems) or glyceraldehyde-3-phosphate dehydrogenase (GAPDH; 1:1000, Abcam) primary antibody at 4°C and then with a secondary antibody. GAPDH was used as a loading control. The protein signaling was developed via Enhanced Chemiluminescence (Solarbio). Relative protein level was analyzed using QuantityOne v4.6 (Bio-Rad) and normalized to GAPDH.

### Deiodinase activity

Homogenates were produced with human liver and skeletal muscle samples homogenized in PE buffers (0.1 mol/L phosphate, 2 mol/L ethylenediamine tetraacetic acid, pH 7.2) using Polytron(Kinematica AG, Lucerne, Switzerland), frozen, and stored at -80°C. D1 activity in liver tissues was measured by incubating 10 µg protein with 0.1 µmol/L (3’, 5’-^125^I) rT3 (100,000 cpm) in 0.1 mL PED10 buffer (PE + 10 mmol/L dithiothreitol (DTT)) for 30 min. D2 activity in skeletal muscles was determined by incubating 200 µg protein with 1 mmol/L (3’, 5’-^125^I) T4 (100,000 cpm) in 0.1 mL PED25 buffer (PE+25 mmol/L DTT) for 1 h. To prevent the labeled T4 substrate from being deiodized by the D3 inner ring, incubation was performed in 0.1 µmol/L unlabeled T3. When 0.1 µmol/L unlabeled T4 is present or absent, it is sufficient to saturate D2. D2 activity is equal to the deiodination of unlabeled T4 minus the deiodination of excess unlabeled T4. The procedure for further determination of ^125^I yield is the same as the D1 determination above. D3 was detected by incubating 100 µg liver protein or 200 µg skeletal muscle protein with 1 mmol/L (3’-^125^I) T3 (200,000 cpm) in 0.1 mL PED50 buffer (PE + 50 mmol/L DTT) for 1 h [31].

### Statistical analysis

G*Power software (ver. 3.1.9.7; Heinrich-Heine-Universität Düsseldorf, Düsseldorf, Germany) was used for efficacy analyses and sample size calculation [32]. SPSS 22.0 was utilized for data analysis.Categorical variables were compared using Fisher’s exact test and presented as frequencies. Continuous variables except age and body mass index (BMI) were analyzed using non-parametric tests. Continuous variables were expressed as mean ± standard deviation or median (interquartile distance (IQR)). Differences between continuous variables were analyzed by t-test or Mann-Whitney U test, with the Spearman correlation coefficient used for correlation analysis. Serum thyroid hormone and IL-1β levels in critically ill patients on the last day of the ICU were used as independent variables. Logistic regression analysis was performed to obtain the predictive probability values, and then the predictive value of serum thyroid hormone and IL-1β levels in patient death was evaluated by receiver operating characteristic (ROC) curve analysis. P < 0.05 indicated a statistical difference.

## Results

### Baseline characteristics

No significant differences were observed in age, sex, BMI, and APACHE II score on the 5th day of the ICU between patients who survived and those who died, and patients who died had longer ICU stays (Table 1).

**Table 1:** Baseline characteristics of patients. Note: The Acute Physiology and Chronic Health Evaluation II (APACHE II) score reflects the severity of illness, with higher values indicating more severe illness.

Parameters	Survivor	Non-Survivor	P
Age (yr)	61.2±15.6	61.7±15.2	0.885
Sex (male/female)	25/15	26/14	0.816
BMI (kg/m^2^)	25.5±5.7	25.8±4.5	0.795
ICU stay (d)	16 (10–28)	11 (7–20)	0.003
APACHE II score on the fifth day of the ICU	11 (7–15)	12 (8–15)	0.702

### Serum thyroid hormones and IL-1β differ between survivors and non-survivors

**Figure 1:**
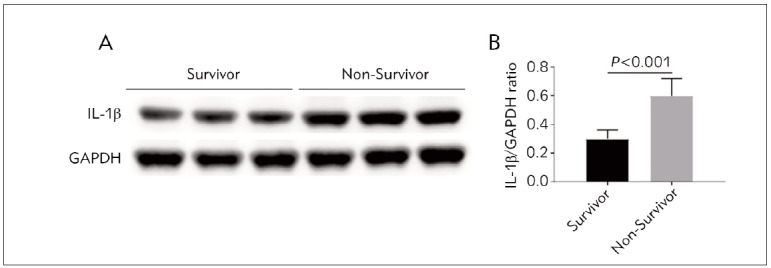
Western blot detection of IL-1β protein expression in peripheral blood mononuclear cells of critically ill patients on the last day of the ICU IL-1β/GAPDH ratio, the relative expression level of IL-1β protein was normalized to GAPDH.

Table 2 shows serum thyroid hormones and IL-1β in surviving and non-survivors. Compared with the survivors, the serum TSH, T4, and T3 were decreased, and the serum rT3 and IL-1β were increased in non-survivors. Moreover, from the 5th day to the last day, TSH, T4, and T3 increased with time, and rT3 and IL-1β decreased in survivors, while TSH, T4, and T3 decreased or remained unchanged, and rT3 and IL-1β increased in non-survivors. IL-1β protein expression was increased in PBMCs of non-survivors on the last day compared with survivors (Figure 1A, B).

**Table 2:** Serum thyroid hormones and IL-1β levels in survivors and non-survivors.

Parameters	Day	Survivor	Non-Survivor	P
TSH (µU/mL)	5	1.21 [0.48–2.26]	0.43 [0.12–1.34]	<0.001
	15	1.46 [0.68–2.45]	0.85 [0.26–2.39]	0.046
	Last day	1.48 [0.76–2.32]	0.45 [0.05–0.96]	<0.001
T4 (µg/dL)	5	5.65 [4.08–7.21]	3.36 [2.47–5.28]	<0.001
	15	6.71 [4.91–8.23]	3.90 [2.88–6.93]	<0.001
	Last day	7.50 [6.11–8.79]	3.39 [2.01–5.45]	<0.001
T3 (ng/dL)	5	74.1 [59.2–92.3]	53.8 [42.1–70.6]	<0.001
	15	87.1 [66.3–107.8]	60.9 [50.5–77.1]	<0.001
	Last day	94.2 [78.0–109.6]	54.4 [42.1–70.0]	<0.001
rT3 (ng/dL)	5	41.0 [28.0–65.7]	59.2 [35.1–87.5]	<0.001
	15	37.5 [25.9–63.4]	63.7 [31.9–101.3]	<0.001
	Last day	33.6 [23.3–54.5]	91.0 [40.3–135.1]	<0.001
IL-1 (pg/mL)	5	6.35 [4.56–8.07]	9.18 [7.30–11.3]	0.001
	15	5.12 [4.05–6.74]	10.6 [8.14–12.5]	<0.001
	Last day	4.07 [3.23–5.48]	12.3 [10.5–13.6]	<0.001

### Predictive value of serum thyroid hormones and IL-1β on the last day of the ICU on patient death

**Figure 2:**
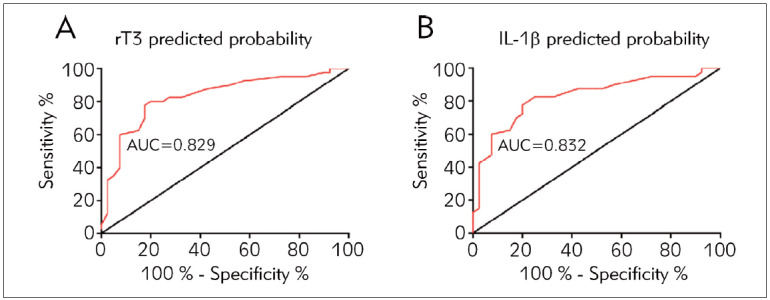
Predictive value of serum thyroid hormones and IL-1β on death in patients on the last day of the ICU.

Serum thyroid hormones and IL-1β levels of critically ill patients on the last day of the ICU were used as independent variables, and logistic regression analysis was performed to obtain the predictive probability values, and then the predictive value for the patients’ deaths was evaluated by using ROC curve analysis. The results showed that serum rT3 and IL-1β had high predictive value (Figure 2A, B).

### Correlation of liver D1 with serum thyroid hormones and IL-1β on the last day of the ICU

**Figure 3:**
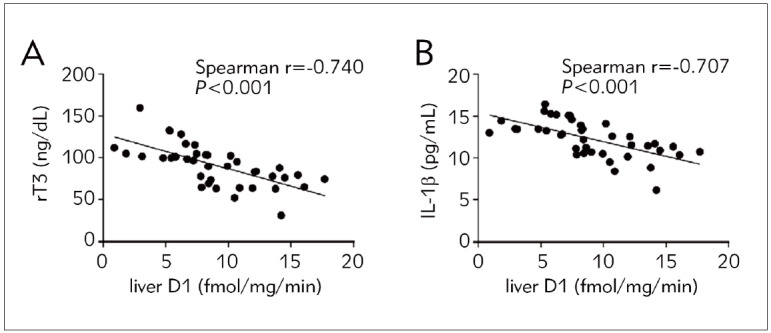
Correlation between liver D1 and serum thyroid hormones and IL-1β on the last day of the ICU.

Spearman’s correlation coefficient was used for correlation analysis. Postmortem liver D1 activity was negatively correlated with serum rT3 and IL-1β on the last day of ICU (Figure 3A, B) but had no correlation with serum TSH, T4, or T3 levels (Table 2).

### Association of liver and skeletal muscle D3 with serum thyroid hormone and IL-1β on the last day of the ICU

**Figure 4:**
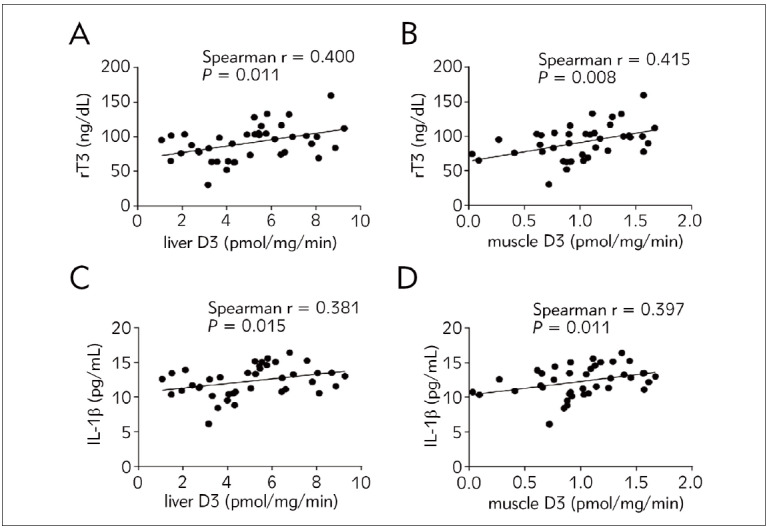
Association of liver and skeletal muscle D3 with serum thyroid hormones and IL-1β on the last day of the ICU.

Postmortem D3 activities were positively associated with serum rT3 and IL-1β levels on the last day of ICU (Figure 4A-D) but not with serum TSH, T4, or T3 levels (Table 3).

**Table 3:** Correlation between tissue deiodinase activity and serum thyroid hormone and IL-1 on the last day of the ICU.

	Liver D1		Liver D3		Muscle D3	
	Spearman r	P	Spearman r	P	Spearman r	P
TSH (µU/mL)	0.212	0.128	-0.235	0.116	-0.257	0.094
T4 (µg/dL)	0.179	0.231	-0.176	0.243	-0.032	0.817
T3 (ng/dL)	0.144	0.332	-0.271	0.075	-0.154	0.309
rT3 (ng/dL)	-0.740	<0.001	0.400	0.011	0.415	0.008
IL-1 (pg/mL)	-0.707	<0.001	0.381	0.015	0.397	0.011

## Discussion

Patients suffering from critical illnesses who require treatment in the ICU uniformly present with alterations in circulating thyroid hormone levels that are referred to by several names such as »nonthyroidal illness syndrome,« »sick euthyroid syndrome,« or »low T3 syndrome« [33] [34]. Decreased serum T3 and elevated rT3 are correlated with disease severity [9], and serum T4 is inversely correlated with mortality [35]. This trial found that from day 5 to the last day of ICU, serum TSH, T4, and T3 increased and rT3 levels decreased with time in survivors, while serum TSH, T4, and T3 levels decreased or unchanged, and serum rT3 levels increased in non-survivors. Serum rT3 on the last day indicated a correlation with post-mortem deiodinase activity.

Patients with significant changes in serum thyroid hormones have higher mortality [36] [37]. This study found significant TSH, T4, and T3 differences between survivors and non-survivors. TSH, T4, and T3 were elevated in surviving patients but not non-surviving patients. From the 5^th^ day to the last day, T4 and T3 continued to increase, and no further TSH was observed after the 15^th^ day, indicating that T4 and T3 both increased with the initial increase of TSH. This is consistent with previous research showing elevated serum TSH leads to elevated T4, marking the beginning of disease recovery [38] [39]. Throughout the ICU period, serum rT3 levels of non-survivors continued to rise. This may be due to the short half-life of rT3 [40] [41], which is a sensitive marker for acute changes in tissue decay-mediated thyroid hormone metabolism during death.

It is estimated that D1 in the liver and kidneys contributes 15–80% of peripheral T3, and D2-containing tissues contribute to the remaining extra-thyroid T3. D1 plays the greatest role in patients with hyperthyroidism, while D2 plays a significant role in patients with hypothyroidism. The decrease in D1 activity will lead to the decrease of T3 production by T4 and the decrease of rT3 clearance [10]. Another possible mechanism for lowering and increasing serum T3 levels is that D1-expressing tissues have lower uptake of T4 and rT3 [14] [15]. This study found that liver D1 activity was negatively correlated with serum rT3 levels.

Under normal conditions, D3 is only present in the liver of the developing fetus and protects the fetus from overexposure to thyroid hormone, indicating that pathological conditions in adulthood may be related to deiodinase changes, especially D3 [42] [43]. D3 is expressed in human skeletal muscle [44]. D3 may reduce skeletal muscle local thyroid hormone levels by converting T4 to rT3 and T3 to 3,3‘-T2. D3 expression in hemangiomas may lead to low T4 and T3 and high rT3 levels [16]. This study found that liver D3 activity positively correlated with serum rT3.

Notably, we were unable to detect any D2 in skeletal muscle samples from these patients, whereas D2 activity was present in skeletal muscle from normal subjects [45]. Elevated serum rT3 concentration may lead to D2 inactivation in critically ill patients [46]. D2 in skeletal muscle promotes the production of serum T3, especially in cases of hypothyroidism [47]. Therefore, skeletal muscle D2 inactivation may also lead to decreased T3 levels in critically ill patients.

Critical illness is related to metabolic and inflammatory disorders [17]. Postoperative serum IL-1β levels are associated with mortality in critically ill patients after major abdominal surgery [18]. In addition, it has been shown that IL-1β inhibits thyroid hormone receptor-β1 gene expression [25], which inhibits hepatic D1 expression [26]. Therefore, we tested serum IL-1β levels in critically ill patients and showed that serum IL-1β levels were elevated in non-survivors compared with the survivors. From day 5 to the last day of ICU, serum IL-1β of survivors decreased with time, while it increased in non-survivors. In addition, IL-1β protein in PMBCs on the last day of non-survivors was increased compared with that of survivors. High levels of IL-1β are associated with abnormal thyroid function [48]. The relationship between deiodinase activity and serum IL-1β was further analyzed. The results showed that liver D1 activity was negatively correlated with serum IL-1β, while liver and skeletal muscle D3 activity was positively correlated with serum IL-1β.

However, this study has some limitations. First, this study only explored the effect of one inflammatory factor, IL-1β, on the prognosis of critically ill patients, and more inflammatory factors need to be included in the study. Second, the present study did not delve into the potential mechanisms by which IL-1β affects deiodinase activity, and it is hoped that this can be further explored in the future.

## Conclusion

This study is the first to explore the relationship between serum thyroid hormones and IL-1β and tissue deiodinase activity. Serum TSH, T4, and T3 levels were decreased compared with survivors, and rT3 and IL-1β were increased in non-survivors. Liver D1 activity was negatively correlated with serum rT3 and IL-1β, while liver and skeletal muscle D3 activities were positively correlated.

## Dodatak

### Acknowledgments

Not applicable.

### Funding

Not applicable.

### Availability of data and materials

The data are available from the corresponding author upon request.

### Ethics statement

This study was approved by the Ganzhou People’s Hospital ethics committee, and the guardian of every subject signed informed consent.

### Authors’ contributions

Z.Z. Zhong designed the research study. Z.Z. Zhong and X.L. Xiao performed the research. X.L. Xiao provided help and advice on the experiments. Z.Z. Zhong and X.L. Xiao analyzed the data.

Z.Z. Zhong wrote the manuscript. All authors contributed to editorial changes in the manuscript. All authors read and approved the final manuscript.

### Conflict of interest statement

All the authors declare that they have no conflict of interest in this work.
